# The Cambridge Experience With Tibial Plateau Fractures in Older Adults: A Case Series

**DOI:** 10.7759/cureus.13311

**Published:** 2021-02-12

**Authors:** Dhiraj Sharma, Azeem Thahir, Vivek Sharma, Matjia Krkovic

**Affiliations:** 1 Department of Trauma and Orthopaedics, Addenbrooke's Hospital, Cambridge University Hospitals NHS Foundation Trust, Cambridge, GBR; 2 Department of Trauma and Orthopaedics, Norfolk and Norwich University Hospitals Foundation Trust, Norwich, GBR

**Keywords:** tibial fracture, tibial fractures, knee injury, comminuted fractures

## Abstract

Complex tibial plateau fractures (TPFs) represent a significant treatment challenge for any Orthopaedic surgeon. Current literature suggests that significantly displaced TPFs in the elderly require operative fixation, an operation that is associated with serious complications including septic and post-operative arthritis. As a result, these patients are five times more likely to require a total knee replacement (TKR). We present a case series of five elderly patients with complex TPFs who made serendipitous recoveries while awaiting operations. Their fractures were deemed so severe that they were being considered for TKR instead of fixation. We discovered their surprising functional improvements while they were being reviewed pre-operatively and decided to delay operating. We are currently unaware of any cases in the literature that have reported such findings. In total, five patients presented in 2019 with closed, varus/valgus stable fractures. They were managed non-operatively in hinged-knee braces, progressively weight-bearing with a minimum follow-up of 10 months. Oxford Knee Scores (OKS) were recorded at zero and four months after their injury. All patients were female with an average age of 69 years. Average fracture depression - 8.48mm, average fracture split - 8.66mm, average OKS reduction - 19%. All patients were able to mobilise independently at four months follow-up. Our results suggest that non-operative management can be considered as primary management in elderly patients with significantly displaced TPFs. Should this fail, or they develop arthritis, a TKR can be performed. This carries two benefits: the patient avoids the significant complications associated with fixation and should a TKR be required, it can more easily be performed in a patient without metalwork in-situ. We feel that the results from this case series might offer insight into a new treatment strategy and continue to closely follow these patients.

## Introduction

Tibial plateau fractures (TPF) make up 8% of fractures in the elderly [1]. Complex TPFs represent a significant surgical challenge and lack strong evidence-based strategies for optimal fixation [2]. In the United Kingdom, the proportion of adults aged over 85 is set to double in 25 years, hence, the long term prognosis of fracture interventions must be closely considered, especially with major weight-bearing joints where effective management will restore mobility and minimise any reduction in quality of life [3,4].

Non-operative treatment should aim to allow for early range of motion to prevent permanent knee stiffness. This can be achieved with casts, bracing and/or early traction. Non-operative management of displaced, unstable tibial plateau fractures is associated with poor outcomes for all Schatzker fractures as demonstrated in Schatzker's seminal paper [5].

Operative management is generally considered in patients with significantly displaced fractures (greater than five millimeter articular step off or condylar widening), those with valgus/varus instability, open fractures and those with few co-morbidities [2]. Typically, these are managed using open reduction internal fixation (ORIF) techniques [2]. Wound breakdown and infection occur commonly as a result of operating on a fragile, contused tissue envelope [6,7]. Additionally, post-operative arthritis renders these patients up to five times more likely to require a total knee replacement (TKR) [8]. As a result, complex TKR is increasingly being considered as a primary management option for TPFs in older adults [9].

We describe a case series of significantly displaced TPFs in older adults who were initially intended to have a TKR but were treated non-operatively with serendipitous results. 

## Case presentation

**Figure 1:**
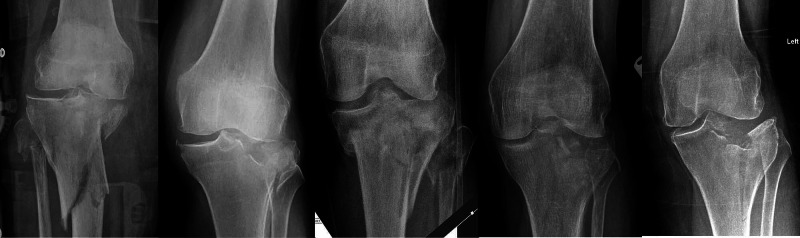
Anterior-posterior radiographs on the day of the injury (Case 1 to 5, left to right)

All patients were American Society of Anaesthesiologists (ASA) grade one or two and prior to the injury were fully ambulatory and independent of their activities of daily living. All fractures were closed without any associated neurovascular injury and were varus/valgus stable. This case series represents every patient treated non-operatively for this injury in 2019, under the care of a single Surgeon who specialises in the management of complex lower limb injuries. Table 1 summarises their mechanism of injury and fracture displacement. Figure 1 demonstrates radiographs taken on the day of injury.

**Table 1:** Patient demographics, mechanism of injury and fracture displacement

Patient	Age (years)	Gender (Male or Female)	Mechanism of injury	Depression (mm)	Split (mm)	Lateral translation (mm)
1	73	Female	Knocked onto knees by a falling tree branch	4.2	8	7.4
2	70	Female	Kicked by a horse	8	5.5	11
3	72	Female	Tripped down four stairs onto hard flooring	4.2	8	8
4	65	Female	Tripped over a dog onto concrete	12	6.8	4
5	64	Female	Collision with a cyclist	14	15	3.8

Treatment

Following the injury, the fractures were reviewed with CT imaging and the Consultant made a management decision that the benefits of TKR outweighed the complications associated with a complex fixation. The patients were managed non-operatively for six weeks to allow for swelling to improve and for the bone to partially heal prior to performing TKR. It was discovered that these patients made good functional recoveries while awaiting their operations so operative management was delayed. Patients were regularly followed up to monitor their recovery. All patients had a minimum clinic follow up of 10 months and retrospectively recorded Oxford Knee Scores (OKS) before the injury and four months after the injury. The OKS is the patient-reported outcomes measure (PROM) evolution tool validated for use by the National Joint Registry, UK [10]. All patients were discussed in knee multi-disciplinary team meetings and treated initially in an above-knee back slab or a cricket-pad splint which was converted to a hinged knee brace within one month. The brace was locked in 10 degrees of flexion and patients were discharged either non-weightbearing or toe-touch weightbearing for six weeks before being advised to gradually fully weight bear as tolerated. Throughout this period of recovery, patients had access to physiotherapy and were initially reviewed every four to six weeks to monitor pain, mobility and clinical/radiographic stability. Venous thromboembolism risk was assessed at every clinic appointment and prescribed accordingly.

Outcomes and follow-up

**Figure 2:**
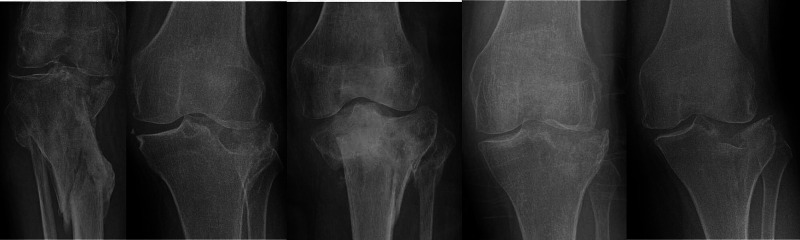
Anterior-posterior radiographs four months following the injury. Case one to five (left to right).

A description of the outcomes for each patient is detailed below. Outcome measures have been summarised in Table 2. Figure 2 contains radiographs taken four months following the initial injury.

**Table 2:** Oxford Knee Scores and range of movement (ROM) four months after the injury

Patient	Oxford Knee Score before injury	Oxford Knee Score 4 months after Injury	ROM (extension – flexion), 4 months after injury
1	45	23	20-50 degrees
2	48	44	10-100 degrees
3	48	40	5-100 degrees
4	48	44	10-110 degrees
5	47	41	0-110 degrees

Case One 

This patient made slow progress using a walking frame for six months, before gradually being able to walk around her house with a frame and use stairs. From six weeks her knee was judged to being clinically stable with satisfactory radiographic alignment. Her OKS reduced by 49% at four months and her range of motion (ROM) was limited between 20-50 degrees of flexion. She was offered a total knee replacement but she was happy with her progress and went on to make a gradual improvement. At 10 months was able to mobilise independently for an hour without pain. 

Case Two 

This patient made good progress, managing to walk independently and ride her horse eight weeks after the injury. Her knee was judged to be clinically stable at 12 weeks with satisfactory radiographic alignment and bone union seen at six months. Her OKS reduced by 8% at four months and her ROM resided at 10-100 degrees of flexion.

Case Three 

This patient initially required an enablement placement for intensive physiotherapy but was in her own home after four weeks. Her knee was judged to be clinically stable with satisfactory radiographic alignment and bone union at six months. At four months she was walking unaided, her OKS had reduced by 17% and her ROM resided at 5-100 degrees of flexion.

Case Four 

This patient made good progression to mobilise fully weightbearing without aids at 12 weeks. Her knee was clinically stable, and radiographs showed satisfactory alignment with bone union seen at six months. Despite 10 degrees of valgus deformity, she was comfortable and happy to continue being treated non-operatively. At four months post-injury her OKS dropped by 8% and her ROM resided at 10-100 degrees of flexion.

Case Five 

This patient made good progress, walking pain-free with only a slight limp at 12 weeks. Her knee was clinically stable, and radiographs showed satisfactory alignment with bone union seen at six months. At four months post-injury her OKS dropped by 13% and her ROM resided at 0-110 degrees of flexion.

## Discussion

Tibial plateau fractures in the elderly present a surgical challenge due to poor bone stock, fracture heterogeneity and a high rate of complications [11]. When Schatzker et al. initially investigated TPFs he concluded that non-operative techniques should be reserved for minimally displaced, stable fractures [5]. At that time, fixation was the only option as TKRs were in their infancy and not a viable back-up operation should initial management fail [12]. 

Despite ORIF being considered the gold standard, it is associated with poor outcomes [6,13]. Biyani et al. looked retrospectively at 32 elderly patients with TPFs treated with ORIF and reported poor outcomes in 12.5% [13]. Infection is a potentially disastrous complication after TPF ORIF and great care and consideration should be made to help prevent it [7]. Young and Barrack reported an infection rate of 88% in their AO type IV fractures, and in those who developed infections, an average of five subsequent surgical procedures were required [14]. Marsh et al. used minimally invasive techniques for complex TPF fixations and 35% of patients required antibiotics and one patient (n=20) developed septic arthritis [15]. Joint incongruity following a fracture is associated with an increased incidence of arthritis, however post-operative arthritis is also a significant complication in those undergoing surgery [16]. As a result of these complications this cohort of patients is five times more likely to require a TKR when compared with a matched cohort [8]. TKR following TPF fixation is more challenging and associated with a higher rate of complications [16]. 

The patients presented in this study had complex fracture patterns. They were discharged home to allow for the fracture to heal and swelling to reduce before being invited back for a TKR. Although we planned TKRs for these patients, they all made serendipitous improvements in their function. Non-operative management led their OKS to drop by an average of 19% at four months. One patient had a significant reduction in her OKS (48%), but 10 months following the injury she was able to walk for an hour unaided without pain. Williams et al. looked at the trends in the OKSs of over 1000 patients undergoing primary TKR for arthritis and reported that at one year post-operatively the average OKS was 34.3 [17]. At four months post-injury, our case cohort reported an average OKS of 38.4. Although the OKS had dropped in our cohort, the drop in function is still better on average than what was seen in the patients presented in Williams' study who had received a TKR for osteoarthritis [17]. This suggests that the drop in OKS associated with the fracture represents a reasonable lowering in function, similar to the scores we would have seen should they have undergone TKR. We acknowledge that our patient cohort may need a TKR in the future and are closely following them up for signs of worsening function.

We present our results in patients who were ASA one or two, with good functioning knee scores pre-injury. We acknowledge that the pre-injury knee scores were made retrospectively and might not represent their exact scores at the time, however, they demonstrate that the patients all had good pre-morbid knee function. We recognise that this treatment strategy should be reserved for patients who are fit and well, with sufficient social care arrangements and as such, recommend multidisciplinary team management with patient involvement at every step. Long periods without weightbearing represents a challenge that not all patients can forgo, so we recommend close follow up and discussing the option for a TKR at every consultation. The short follow-up period is a limitation of this study. We acknowledge that these patients are at higher risk of requiring a TKR in the future, however, we plan to closely evaluate their progress. 

## Conclusions

Based on our case series we propose that contrary to the current vogue, elderly patients with significantly displaced TPFs can be managed initially without an operation. This avoids subjecting patients to an ORIF, which is associated with significant complications, and potentially gives patients the chance to avoid an operation altogether. This cohort of patients is more likely to require a TKR regardless of if they are treated with fixation or not. As techniques for TKR have become more advanced, it is a good surgical option that can be reserved should non-operative management fail. A reduction in OKS in these patients is expected, but further evaluation of long-term outcomes is required to more accurately guide management. We aim to evaluate whether this treatment strategy is acceptable to patients in the long-term, whether it avoids the need for a TKR and how long it might delay the patient from requiring a TKR. 
